# LRR-RLK family from two *Citrus* species: genome-wide identification and evolutionary aspects

**DOI:** 10.1186/s12864-016-2930-9

**Published:** 2016-08-12

**Authors:** Diogo M. Magalhães, Larissa L. S. Scholte, Nicholas V. Silva, Guilherme C. Oliveira, Cyril Zipfel, Marco A. Takita, Alessandra A. De Souza

**Affiliations:** 1 Instituto Agronômico, Centro de Citricultura Sylvio Moreira, Cordeirópolis, São Paulo Brazil; 2 Departamento de Genética e Biologia Molecular, Universidade Estadual de Campinas, Campinas, São Paulo Brazil; 3 Instituto Nacional de Ciência e Tecnologia em Doenças Tropicais, Grupo de Genômica e Biologia Computacional, Centro de Pesquisas René Rachou, Fundação Oswaldo Cruz, Belo Horizonte, Minas Gerais Brazil; 4 Instituto Tecnológico Vale – ITV, Belém, Pará Brazil; 5 The Sainsbury Laboratory, Norwich Research Park, Norwich, NR4 7UH UK

**Keywords:** *Citrus sinensis*, *Citrus clementina*, Leucine-rich repeat receptor-like kinase, Phylogeny

## Abstract

**Background:**

Leucine-rich repeat receptor-like kinases (LRR-RLKs) represent the largest subfamily of plant RLKs. The functions of most LRR-RLKs have remained undiscovered, and a few that have been experimentally characterized have been shown to have important roles in growth and development as well as in defense responses. Although RLK subfamilies have been previously studied in many plants, no comprehensive study has been performed on this gene family in *Citrus* species, which have high economic importance and are frequent targets for emerging pathogens. In this study, we performed *in silico* analysis to identify and classify LRR-RLK homologues in the predicted proteomes of *Citrus clementina* (clementine) and *Citrus sinensis* (sweet orange). In addition, we used large-scale phylogenetic approaches to elucidate the evolutionary relationships of the LRR-RLKs and further narrowed the analysis to the LRR-XII group, which contains several previously described cell surface immune receptors.

**Results:**

We built integrative protein signature databases for *Citrus clementina* and *Citrus sinensis* using all predicted protein sequences obtained from whole genomes. A total of 300 and 297 proteins were identified as LRR-RLKs in *C. clementina* and *C. sinensis*, respectively. Maximum-likelihood phylogenetic trees were estimated using *Arabidopsis* LRR-RLK as a template and they allowed us to classify *Citrus* LRR-RLKs into 16 groups. The LRR-XII group showed a remarkable expansion, containing approximately 150 paralogs encoded in each *Citrus* genome. Phylogenetic analysis also demonstrated the existence of two distinct LRR-XII clades, each one constituted mainly by RD and non-RD kinases. We identified 68 orthologous pairs from the *C. clementina* and *C. sinensis* LRR-XII genes. In addition, among the paralogs, we identified a subset of 78 and 62 clustered genes probably derived from tandem duplication events in the genomes of *C. clementina* and *C. sinensis*, respectively.

**Conclusions:**

This work provided the first comprehensive evolutionary analysis of the LRR-RLKs in *Citrus*. A large expansion of LRR-XII in *Citrus* genomes suggests that it might play a key role in adaptive responses in host-pathogen co-evolution, related to the perennial life cycle and domestication of the citrus crop species.

**Electronic supplementary material:**

The online version of this article (doi:10.1186/s12864-016-2930-9) contains supplementary material, which is available to authorized users.

## Background

Signaling through cell surface receptors is essential for cells to communicate and interact with the environment. Plant cells are able to perceive and transduce a wide range of signals mainly through receptor-like kinases (RLKs), which mediate cell-to-cell communication by binding to extracellular ligands or forming heteromeric complexes to mediate intracellular signaling [1]. These modular kinase receptors belong to a large monophyletic gene family with more than 400 members identified in *Arabidopsis* [2]. RLKs are typically transmembrane (TM) proteins with a variable amino-terminal extracellular domain (ECD) and a conserved cytoplasmic serine/threonine kinase domain (KD) in the carboxyl-terminal region [3]. The ECD regions play important roles in the recognition of internal signals or environmental stimuli and, according to their features, can be used to classify RLKs [4]. More than 21 structural classes were classified in *Arabidopsis* RLKs based on their ECDs, with the largest one containing leucine-rich repeats (LRRs) [2]. Phylogenetic-based analysis of the *Arabidopsis* RLKs using the KDs and structural comparison of their ECDs allowed the identification of more than 40 subfamilies [2].

In plants, LRR-RLK proteins constitute a diverse group of transmembrane receptors involved in many biological functions usually associated with growth and development [5–9] and responses to biotic and abiotic stresses [10–13]. More than 200 LRR-RLK genes have been identified in the fully sequenced *Arabidopsis* genome [14–16]. Concerning plant-microbe interactions, certain LRR-RLKs play essential roles in defense responses to pathogen attacks by recognizing conserved pathogen- or microbe-associated molecular patterns (PAMPs/MAMPs) such as flagellin and elongation factor thermo unstable (EF-Tu) [17, 18]. LRR-containing proteins are suitable for pathogen recognition because their structural plasticity allows them to bind to many distinct ligands, such as proteins, peptides or lipids [19]. Receptor proteins that are able to recognize PAMPs/MAMPs are designated pattern-recognition receptors (PRRs) [17] and represent an essential step for the host to perceive and defend itself against pathogens by triggering innate immune responses. Many PRRs belong to the LRR-RLK subfamily [18, 20]. The *Arabidopsis* FLAGELLIN SENSING 2 (FLS2) [21], EF-TU RECEPTOR (EFR) [22] and rice XA21 [23] are among the best-studied plant PRRs and can activate immunity responses by perceiving specific bacterial proteins (or derived peptidic epitopes). These well-characterized PRRs belong to the XII group of LRR-RLKs (LRR-XII), suggesting an important role in mediating immunity responses during plant-microbe interactions.

*Citrus* comprise some of the most economically important crops in the world, and the species of this group produce fruits with great commercial value, such as oranges, mandarins, lemons, grapefruits and pummelos. While *Citrus clementina* represents one of the major species of mandarins, consumed as fresh fruit [24], sweet orange (*C. sinensis*) has the largest commercial importance, mainly due to the orange juice market [25]. The genomes of these species were recently sequenced and even though the identity and contribution of ancestors in the composition of the domesticated citrus genome was unclear, it is suggested that these crops are hybrids derived mainly from *C. maxima* and *C. reticulata* [26, 27]. *Citrus* species are mostly diploid and display a basic chromosome number of x = 9. Substantial segmental synteny is observed with other eudicots and an orthology relation of one to one across oranges and plants such as grape, strawberry and cacao suggests the inexistence of recent whole genome duplication (WGD), with the exception of a triplication genome shared by all eudicots [26]. *Citrus* is part of the Sapindales order, a sister order of Brassicales into the Malvidae family, which allows the performance of studies involving genomic comparisons with *Arabidopsis thaliana* [26].

The main problem that affects the citrus culture worldwide is the huge amount of phytopathogens [24], which cause significant damage to the citrus agribusiness. Apomictic reproduction, high juvenility and a long cultivation period are characteristics that contribute to a narrow genetic diversity in citrus crops, which increases the probability of the appearance of diseases and makes it difficult for breeding programs to obtain materials with increased resistance to pathogens [27]. Although there is narrow genetic diversity, there are different levels of resistance among *Citrus* species for different diseases, such as the Citrus canker [27–29], Alternaria brown spot [30], Huanglongbing [31] and Citrus variegated chlorosis (CVC) [32, 33]. *Xylella fastidiosa*, for instance, causes CVC in all commercial sweet orange varieties, but not in *C. clementina*, despite both species being derived from the same ancestors [34]. The comparison of defense gene families among plants with different levels of resistance is a strategy for better understanding the host defense in plant-pathogen interactions. Considering the recent sequencing of the complete genomes of *C. clementina* and *C. sinensis* and the involvement of LRR-RLKs in defense responses, we performed *in silico* analyses to elucidate and compare the structural organization of LRR-RLK members from the *Citrus* databases. The LRR-RLK subfamily has been characterized in plants such as *Arabidopsis*, rice, *Populus*, tomato, and others [35–39], but no comprehensive study was performed for *Citrus* species.

## Results and discussion

### Identification of *Citrus* LRR-RLKs

To identify the LRR-RLK subfamily members encoded by *C. sinensis* and *C. clementina* genomes, we used a combined computational approach. The predicted proteomes of each *Citrus* species and *A. thaliana* were used as inputs (Table 1) to build signature databases with the *InterProScan* tool. The resulting data were then uploaded into relational databases.

**Table 1:** Genome data of *C. clementina, C. sinensis*, and *A. thaliana*

	Plant species		
	*Citrus clementina*	*Citrus sinensis*	*Arabidopsis thaliana*
Database version	clementina 1.0 (version 1.0)	CsiDB 2013 (version 2.0)	TAIR 10 release
Estimated genome size	301.4 Mb	367 Mb	129 Mb
Protein-coding loci	24,533 sequences	29,445 sequences	27,416 sequences
Alternative transcripts	9,396 sequences	14,982 sequences	4,693 sequences
Total transcripts	33,929 sequences	44,427 sequences	32,109 sequences
Available on	https://phytozome.jgi.doe.gov/pz/portal.html	http://citrus.hzau.edu.cn/orange/	http://arabidopsis.org/

A search for protein sequences with both kinase and transmembrane signatures was initially performed for the identification of surface RLK family homologs. The catalytic KD was detected in 1,169, 1,208, and 1,034 non-redundant protein sequences from *C. clementina, C. sinensis* and *A. thaliana*, respectively. Plant protein kinases are one of the largest existing gene families and represent approximately 4 % of all coding genes in *A. thaliana* [40]; a similar number was found for *C. clementina* (4.9 %) and *C. sinensis* (4.0 %). These percentages of genes encoding kinase proteins are close to what was found in poplar and rice [41]. Among these protein sequences, 617, 626 and 466 members of *C. clementina*, *C. sinensis* and *A. thaliana*, respectively, contained the KD and TM helices (Table 2). In the present study, we did not work with alternative splicing variants, and we considered only the membrane surface RLKs in our analysis, which did not include the receptor-like cytoplasmic kinases (RLCKs) because they do not have TM domains [38, 42]. For *A. thaliana*, approximately 620 RLK members have been reported to be present in the genome, including almost 150 RLCKs [4]. Thus, the number of cell surface RLKs identified for Arabidopsis in this work (466) is consistent with previous reports [16]. Cell surface RLKs displaying LRR-type ECDs were considered putative LRR-RLK subfamily members. LRR-RLKs belong to a large subfamily for which we identified 300, 297 and 236 protein sequences in the *C. clementina, C. sinensis* and *A. thaliana* genomes, respectively, which represents approximately 50 % of the total surface RLKs in each genome. We also removed the *A. thaliana* sequences that were the products of alternative splicing, as previously performed for *C. clementina* and *C. sinensis*. The result was compared to an *A. thaliana* LRR-RLK dataset [14, 15] to improve accuracy. Thus, from the 236 *A. thaliana* protein sequences, we considered a final dataset containing 209 LRR-RLK sequences for further analysis (Table 2; Additional file 1). The KDs from LRR-RLKs of *C. clementina, C. sinensis* and *A. thaliana* were identified by searching for diagnostic domains according to the functional annotation in the Pfam database (Pkinase_Tyr PF07714 and Pkinase PF00069). As reported by Shiu et al. [38], some proteins exhibited more than one catalytic KD. In these cases, we further analyzed the catalytic domains to determine whether the truncated regions should be manually merged or kept as different KDs. After another round of filtering, such as removing gap-rich regions and excluding redundant sequences, the final dataset used for the evolutionary analyses contained a total of 302 and 304 KDs from *C. clementina* and *C. sinensis*, respectively, in addition to the 209 KDs from the *A. thaliana* LRR-RLKs (Table 2; Additional file 2).

**Table 2:** Protein classification according to the presence of diagnostic domains in *C. clementina, C. sinensis*, and *A. thaliana* proteomes

Predicted proteins	Plant species		
	*C. clementina*	*C. sinensis*	*A. thaliana*
Kinases	1,208	1,169	1,034
TM kinases	617	629	466
TM kinases with LRR (KD)^a^	300 (302)	297 (304)	209 (209)

^a^The numbers in parenthesis represent the total number of kinase domains identified in the TM kinases with LRR

### Evolutionary analyses and structural organization of LRR-RLKs

**Fig. 1:**
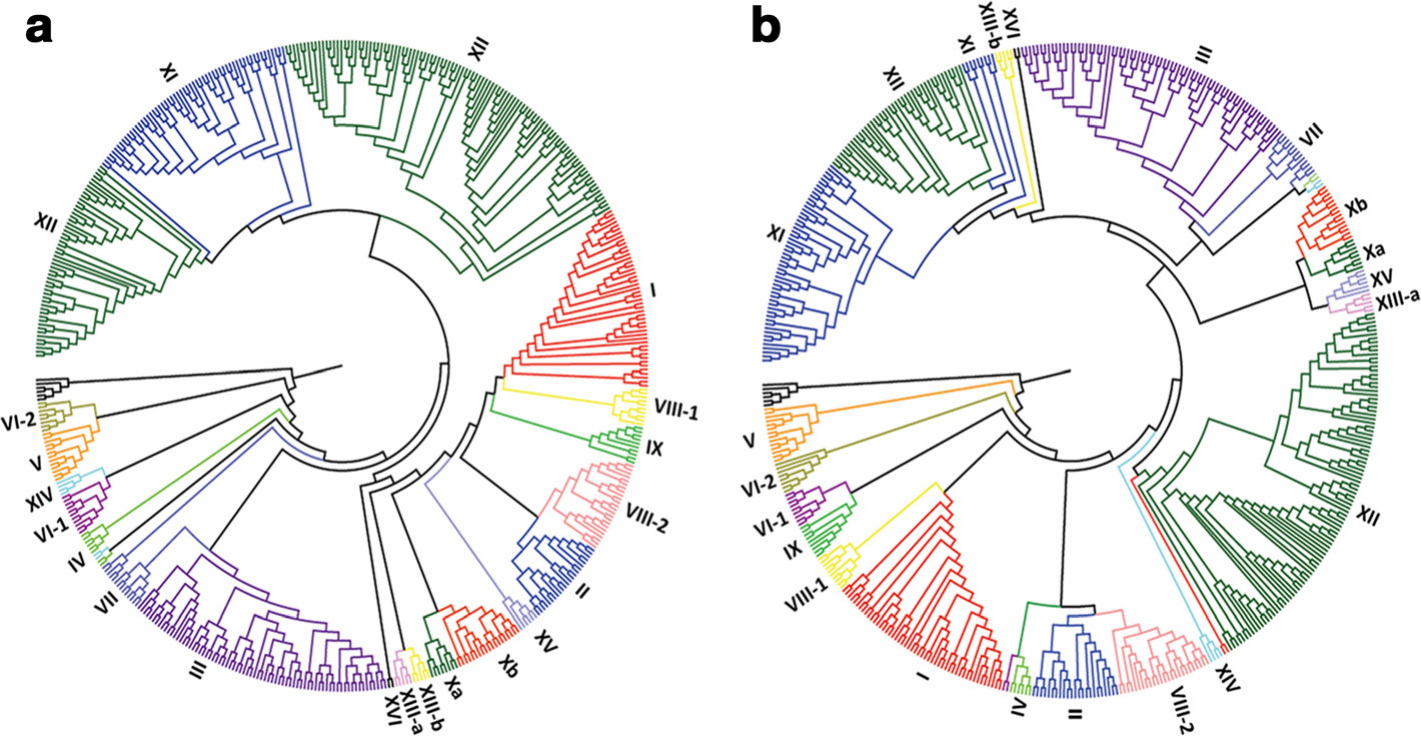
Phylogenetic trees of LRR-RLK from *Citrus clementina* (**a**) and *Citrus sinensis* (**b**). The phylogenetic trees were established with amino acid sequences from kinase domains with the Maximum-likelihood method. The numbers refer to each LRR-RLK subfamily (I-XVI)

An identification and classification of LRR-RLK from *C. clementina* and *C. sinensis* was previously done using genome assemblies obtained from the outdated Phytozome v.7 [41]. In this work we used currently accepted genome assemblies to identify,classify and perform a comprehensive genomic study for the LRR-RLK subfamily groups in the *C. clementina* and *C. sinensis*. The KD sequences from each *Citrus* dataset were independently aligned with the KDs from all *A. thaliana* LRR-RLK subfamily members. Maximum-likelihood (ML) phylogenetic trees allowed us to estimate the evolutionary relationships among the sequences (Fig. 1). The *Citrus* sequences that clustered together with known members of *A. thaliana* LRR-RLK were defined as part of the correspondent group. The majority of clades in each phylogeny was well-supported with confidence statistical values above 0.70 (Additional files 3 and 4), demonstrating the reliability of the generated data (Fig. 1). The robustness of our analysis was confirmed by generating another phylogenetic tree using the LRR-RLKs from both *Citrus* species together in the same tree (Additional file 5). Of 606 KDs, 601 (>99 %) were grouped in well-supported clades, as observed in the individual analysis of the *C. clementina* and *C. sinensis* datasets (Fig. **1**), reinforcing the hypothesis that these sequences were evolutionarily related.

The topology of ML phylogenetic trees allowed us to distinguish, in the *C. clementina* and *C. sinensis* genomes, the same 16 groups of LRR-RLKs (I to XVI) previously described for *A. thaliana* [14] that were used for *Citrus* classification (Table **3**; Additional file 6). Additionally, group XII, which was the focus of our work, presents the characteristic division in two sub-groups (Fig. 1; Additional file 5) as also reported for other plant species [15, 37].

**Table 3:** Total number of receptors distributed in the different groups of LRR-RLKs

Groups	Plant species		
	*C. clementina*	*C. sinensis*	*A. thaliana*
LRR I	9	11	38
LRR II	10	10	13
LRR III	32	33	41
LRR IV	4	5	4
LRR V	4	5	9
LRR VI-1	6	5	6
LRR VI-2	4	4	4
LRR VII	6	5	8
LRR VIII-1	3	3	7
LRR VIII-2	12	14	12
LRR IX	6	5	4
LRR Xa	4	4	4
LRR Xb	10	8	9
LRR XI	30	33	29
LRR XII	148	140	9
LRR XIII-a	2	2	3
LRR XIII-b	2	2	3
LRR XIV	3	3	3
LRR XV	4	4	2
LRR XVI	1	1	1
Total	300	297	209

In general, the number of LRR-RLK receptors for most of the subfamily groups among the *Citrus* species was similar to *A. thaliana,* except for two of them, i.e., LRR-I and LRR-XII. Regarding LRR-I, 38 members were reported for *A. thaliana,* while in *Citrus,* we identified only 9 and 11 members for *C. clementina* and *C. sinensis*, respectively. Despite having a smaller genome [43], *A. thaliana* had about four times more RLKs in this group compared to the *Citrus* species. Although *A. thaliana* LRR-I includes receptor proteins associated with defense responses, such as IMPAIRED OOMYCETE SUSCEPTIBILITY 1 (IOS1) [44] and FLG22-INDUCED RECEPTOR-LIKE KINASE 1 (FRK1) [45], the majority of members in this group has unknown functions. According to Fischer et al. [39], the last common ancestor of angiosperms (LCAA) probably had only 7 LRR-I in the genome and the expansion rate was related to ancestral expansion rather than species-specific events, suggesting that the high copy number found in *A. thaliana* was due to Brassicaceae expansion and subsequent retention in this species. For *Citrus*, in contrast, the number of LRR-I remained the same as suggested by LCAA, perhaps because no recent WGD was detected in *Citrus* plants [26].

In relation to LRR-XII, *C. clementina* and *C. sinensis* showed 148 and 140 members, respectively, while in *A. thaliana* there were only 10 corresponding members. However, one of them (At2g24130) was not included in our analysis because it did not show a predicted transmembrane helix domain in TMHMM v 2.0. If again we compare this with the number of receptors in LCAA LRR-XII (13 genes) [39], it is possible to verify a slight reduction of this group in Arabidopsis, while for *Citrus* species, the LRR-XII had a stronger numerical expansion. Interestingly, as observed for *C. clementina* and *C. sinensis*, the LRR-XII group of different plant species also underwent an expansion [38, 39]. The dynamic rates of duplication, retention and loss of genes occurred independently in each subgroup of LRR-RLKs, which resulted in a distinct composition between species, for example, related to LRR-I and XII (Table 4). *A. thaliana* LRR-XII contains two of the most studied PRRs, i.e., FLS2 and EFR, which are involved in the perception of the bacterial PAMPs flagellin and EF-Tu, respectively [21, 22]. In addition to EFR and FLS2, the LRR-RLK XII XA21 from *Oryza longistaminata* is another important well-studied PRR [23], which recognizes the bacterial sulfated protein RaxX [46].

**Table 4:** Total number of LRR-RLKs, LRR-XII and LRR-I found in different plant species

Plant species	LRR-RLK	LRR-XII	LRR-I	Reference
*LCAA*	150	13	7	[39]
*Glycine max*	467	73	23	[69]
*Populus trichocarpa*	379	42	33	[36]
*Brassica rapa*	303	25	36	[70]
*Solanum lycopersicum*	256	54	8	[37]
*Oryzae sativa*	384	127	42	[38]
*Theobroma cacao*	253	63	12	[71]
*Arabidopsis thaliana*	209	9	38	This work
*Citrus clementina*	300	148	9	This work
*Citrus sinensis*	297	140	11	This work

The expansion or reduction in the size of gene families is a result of evolutionary events usually related to duplications, *de novo* creation of genes and deletions [47]. Selective pressure to perceive changing environment signals can drive the expansion of specific RLK subfamily groups that may have important functional effects related to adaptation, for example, to fast-evolving pathogens [14, 41]. It was reported that LRR-XII is a group that keeps expanding and their members are involved in biotic stress responses [39]. In general, we observed that in *Citrus* and other crop species, the number of LRR-XII is increased in relation to LCAA (Table 4), suggesting that domestication may be contributing to the expansion of this group.

### Evolutionary aspects of *C. clementina* and *C. sinensis* LRR-XII

#### Analysis of LRR-XII orthologs

Based on the large expansion of *Citrus* LRR-XII and its important role in response to biotic stresses, we further focused on homology studies involving this receptor group. Initially, we searched for orthologs through integrated analyses of phylogeny, sequence similarity and chromosomal distribution in the *C. clementina* and *C. sinensis* LRR-XII subfamily.

**Fig. 2:**
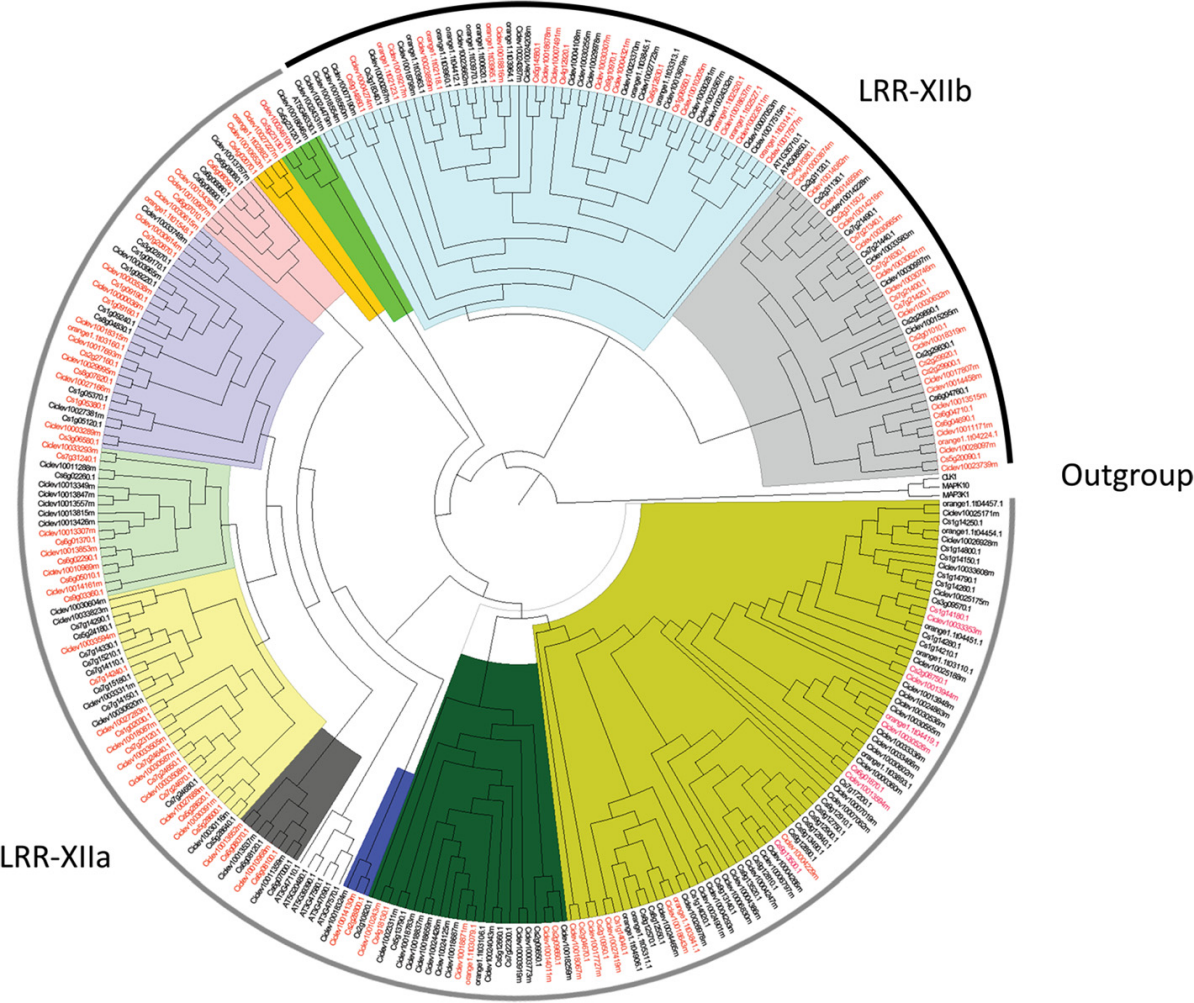
Phylogenetic tree of LRR-XII from *C. clementina*, *C. sinensis* and *A. thaliana*. The phylogenetic tree was established with full sequences using the Maximum-likelihood method. Different colors represent the 13 clades that were identified. Red sequences indicate the orthologous pairs of *C. clementina* and *C. sinensis*. Rooting of the tree was conducted with an outgroup, which was formed by human kinase sequences, a mitogen-activated protein kinase kinase kinase 1 (M3K1), dual specificity protein kinase (CLK1) and mitogen-activated protein kinase 10 (MK10)

Understanding evolutionary aspects, such as paralogy and orthology relationships between genes, is important to deduce the evolution of species [48]. The clades of the phylogenetic tree formed by *C. clementina* and *C. sinensis* LRR-XII sequences in association with the Bidirectional Best Hits (BBH) method were used to detect the LRR-XII orthologs. A total of 68 paired sequences were identified whose similarity was confirmed through 13 well-supported clade grouping of the sequences from the reconstructed phylogenetic tree (Fig. 2; Additional file 7).

The tree topology allowed us to distinguish two monophyletic groups, one formed by two clades (light blue and light gray), related to the LRR-XIIb members and the other formed by the remaining eleven clades, which represent the LRR-XIIa (Fig. **2**), as can also be observed in Fig. **1**. LRR-XIIa harbors seven of the nine members from the previously assigned *A. thaliana* LRR-XII group. LRR-XIIb harbors the two remaining members of the group, At1g35710 and At4g08850, indicating a non-monophyletic origin for the LRR-XII subfamily. This distinct grouping of LRR-XII members was also observed in phylogenetic analysis using tomato [37] and *A. thaliana* [15].

The two *Arabidopsis* members mentioned above and the correspondent *Citrus* members from the same clade (light gray and light blue in Fig. 2), did not share enough similarity with the other LRR-XII members. Previous work showed that these two members fell in the LRR-XI group [15] and they should comprise an independent group of LRR-RLKs. Based on this approach, the resulting LRR-XII group from *C. clementina* and *C. sinensis* would comprise 123 and 126 members, respectively.

### LRR-XII kinase RD motif analysis

**Fig. 3:**
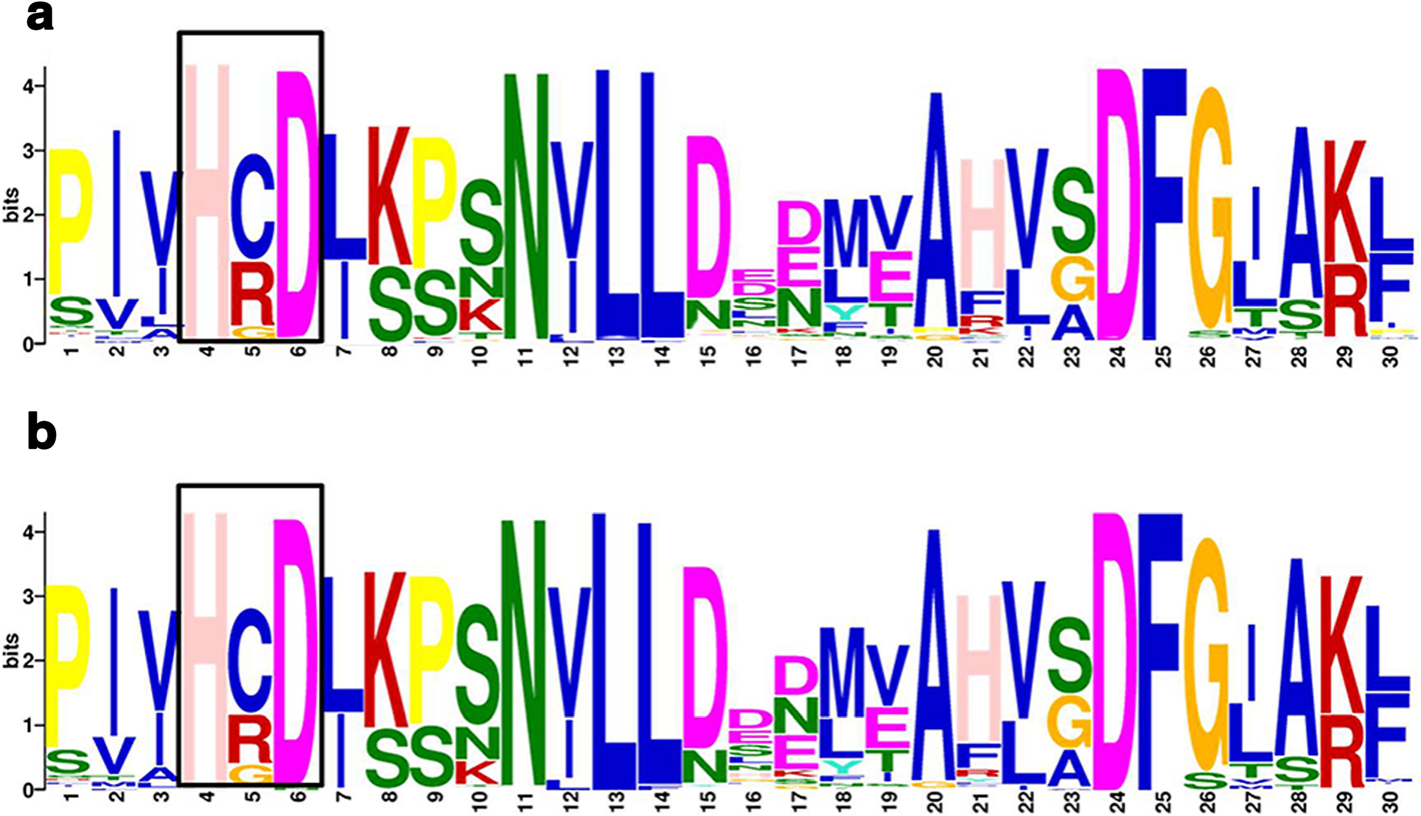
Activation loop region from the *C. clementina* (**a**) and *C. sinensis* (**b**) kinase domain of the LRR-XII proteins. The represented region refers to the conserved amino acid in the activation loop with the majority of the sequences showing absence of arginine (R) in the RD motif (box)

Non-arginine-aspartate (non-RD) kinases are kinases that lack the highly conserved arginine (R) that precedes the catalytic aspartate (D), which is typical of most kinases [49]. This subclass of kinases is often found as part of pattern recognition receptors [50, 51]. Considering the high incidence of pathogens that cause diseases to *Citrus* and their importance in the recognition of conserved microbial patterns, it is important to identify these classes of kinase proteins in the *C. clementina* and *C. sinensis* LRR-XII groups. A total of 93 of 148 and 94 of 140 LRR-XII elements were classified as non-RD in *C. clementina* and *C. sinensis*, respectively (Additional file 8), which represents approximately 70 % in both *Citrus* LRR-XII groups. Usually, non-RD kinases carry the cysteine (C) or a glycine (G) amino acid residue in the substitution of the highly conserved arginine (R) in the HRD motif [50] and the same is observed for both *Citrus* species, in which non-RD carrying C or G in place of R accounts for over 95 % (Fig. 3). However, in a few cases, tryptophan (Y) or serine (S) substitutes for R (less than 2 %).

These changes can affect the charge of the molecules and potentially modify the kinase regulatory and catalytic mechanisms [50]. Only 7 members from *C. clementina* and 9 members from *C. sinensis* have a kinase with an alternative catalytic function (ACF), which did not show either RD or non-RD motifs. The non-RD kinases identified in *Citrus* LRR-XII open new possibilities for further studies involving the function of these receptors in defense responses by recognition of microbial signatures.

In addition to the identification of RD and non-RD kinases in the *Citrus* LRR-XII group, we analyzed the LRR-XII RD and non-RD kinase motifs in *A. thaliana*. Only two sequences showed the RD motif, while all the other seven were non-RD kinases, as already identified by other authors [15, 50]. These two RD kinases correspond to RLK members, which were grouped in a separated clade of the phylogenetic trees (At4g08850.1 and At1g35710.1) (Fig. 2). Additionally, all the non-RD kinases were grouped in the clade that contained LRR-XIIa (Fig. 2). Curiously, and in agreement with this classification, 98 % and 100 % of the RD members from *C. clementina* and *C. sinensis*, respectively, were grouped across LRR-XIIb. For the non-RD kinases, 97 % and 99 % from *C. clementina* and *C. sinensis*, respectively, were grouped across LRR-XIIa. This separation of the *Citrus* RD and non-RD kinases in two distinct clades, as observed in *A. thaliana*, suggests a possible common phylogenetic origin for each of the RD and non-RD kinases in the LRR-XII group.

### LRR-XII tandem duplication paralogs in *C. clementina* and *C. sinensis*

We analyzed the paralogous sequences in the *C. clementina* and *C. sinensis* LRR-XII group because they can evolve new functions in relation to the ancestor proteins [52] (Additional files 9 and 10).

**Fig. 4:**
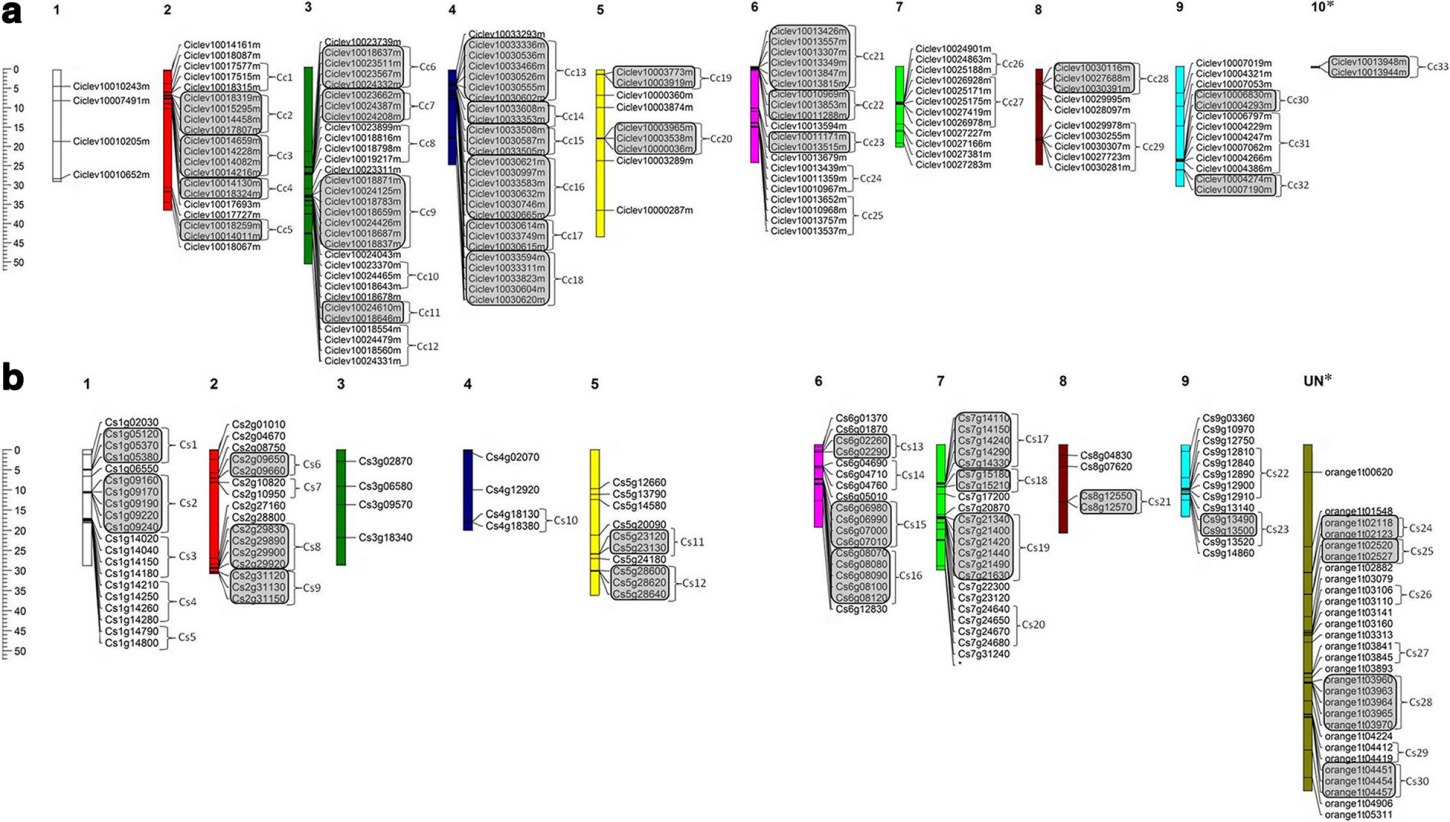
Chromosomal distribution of LRR-XII from *Citrus*. LRR-XII genes were mapped in the chromosomes of *Citrus clementina* (**a**) and *Citrus sinensis* (**b**). Highlighted areas correspond to probable duplication blocks. Cc and Cs represents gene clusters within 200 kb genomic regions in *C. clementina* and *C. sinensis*, respectively. * Chromosomes 10 or UN are not real chromosomes. They were composed by sequences that were not assembled in the 9 correct chromosomes

We identified 78 and 62 LRR-XII tandem duplicated sequences in the genomes of *C. clementina* and *C. sinensis*, respectively. Because both species are hybrids derived from a cross of *C. reticulata* and *C. maxima*, it is difficult to make any assumptions about when, in the evolutionary history of the group, these duplications appeared, even though it is known that they have the same parents [27]. An analysis of the chromosomal distribution of LRR-XII allowed us to detect tandem duplications of this gene family on the chromosomes from *C. clementina* (Fig. **4**a) and *C. sinensis* (Fig. 4b).

The *Citrus* LRR-XII receptors are distributed in all chromosomes but are mainly concentrated on chromosome 3 for *C. clementina* and on chromosome Un (for unassigned contigs) for *C. sinensis*. Chromosomes 4 and Un from *C. clementina* and *C. sinensis*, respectively, displayed the majority of the tandem duplicated genes. Duplication events seem to be pronounced in domesticated plant species [53]. Segmental duplication events must have contributed to the acquisition of novel and distinct functions in relation to the ancestor by neo-functionalization or sub-functionalization [54]. Considering the large number of pathogens in citrus crops, the observed large expansion of *Citrus* LRR-XII might be regarded as a plant-specific adaptation to extracellular signal perception, for example, to recognize different PAMPs [16].

### Identification and distribution of LRR-XII gene clusters

Gene duplication is an important strategy for adaptive evolution in plants [55]. To identify clusters that encompass LRR-XII tandem duplicated genes, we grouped these genes in each *Citrus* genome into the same cluster if they were arranged in a genomic fragment with a maximum of 200 Kb. LRR-XII gene clusters are present in all chromosomes, with the exception of chromosomes 1 and 3 from *C. clementina* and *C. sinensis*, respectively (Fig. 4; Additional file 11). A distribution analysis revealed 117 of 148 LRR-XII genes of *C. clementina* (79 %) were found in 33 cluster regions, and for *C. sinensis*, 94 of 140 LRR-XII genes (67.1 %) were distributed in 30 clusters. Tandem duplications seem to be an important mechanism for expansion of the LRR-XII group, which could be confirmed by the presence of the LRR-XII gene in clusters. Approximately 70 % and 63 % of these clusters are formed by tandem duplicated paralogs in the *C. clementina* and *C. sinensis* genomes, respectively. Wang et al. [56] also demonstrated high clustering and the importance of duplication events in the expansion of *Citrus* nucleotide binding site (NBS) genes, which is a large class of intracellular immune receptor genes that also display LRR domains beyond the nucleotide-binding site domain. Clustering in NBS gene loci has been reported in many species, including Arabidopsis and rice [55]. On the other hand for LRR-XII genes, this expansion is not widespread in plants as the NBS genes are mainly observed in rice [57] and citrus.

Of the 68 LRR-XII orthologous pairs identified for *C. clementina* and *C. sinensis*, 46 and 38 genes from *C. clementina* and *C. sinensis*, respectively, were located in cluster regions (Additional file 7). We identified orthologous pairs in the same clusters, which suggested high conservation and correspondence of these clusters between *C. sinensis* and *C. clementina* genomes. These data suggested blocks of elevated homology among *C. clementina* and *C. sinensis* LRR-XII sequences and chromosome regions.

### Syntenic blocks in LRR-XII and *Citrus* genomes

**Fig. 5:**
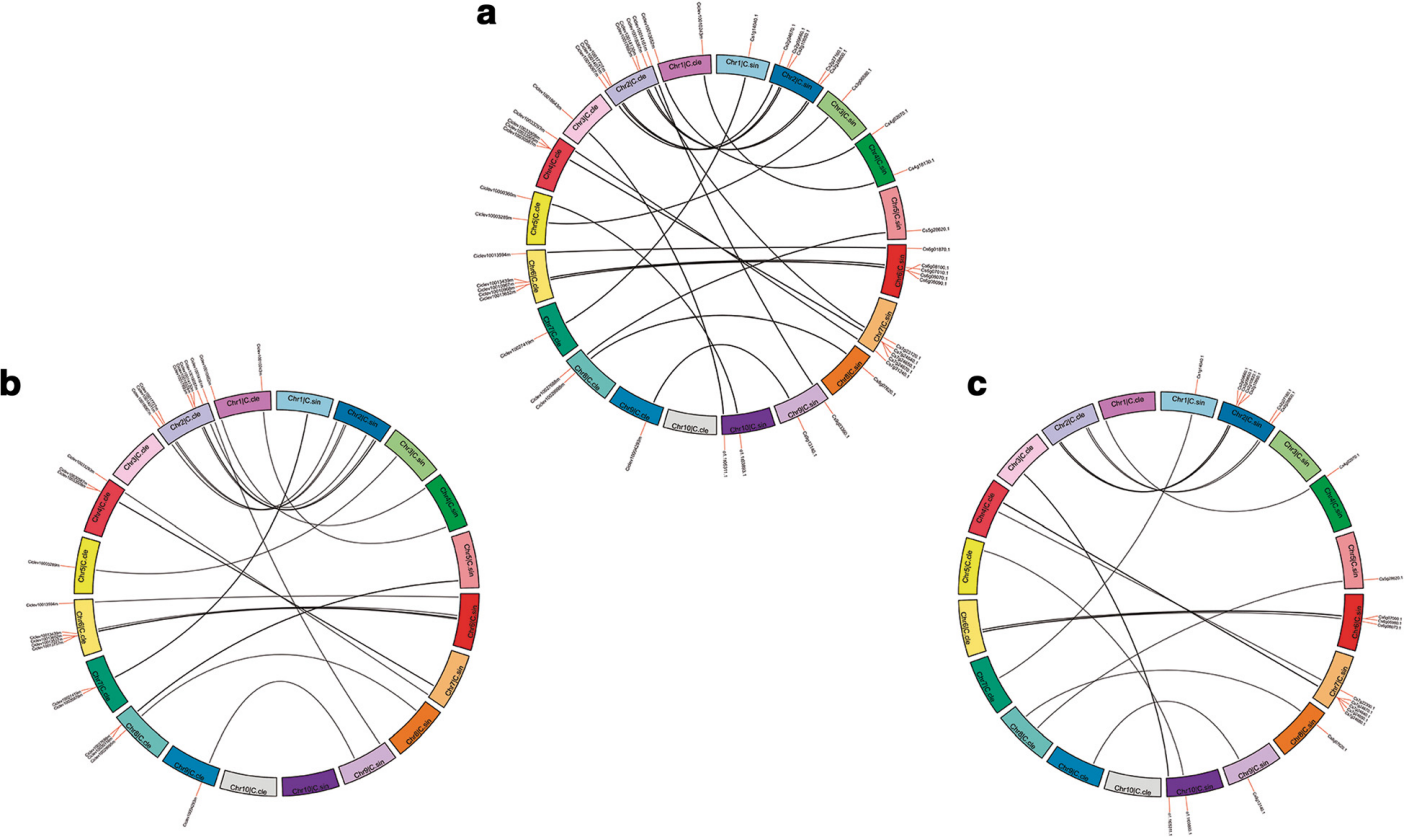
Synteny analysis. Genome collinearity between *C. clementina* and *C. sinensis* LRR-XII (**a**). Independent homology of LRR-XII genes and SBs in the genomes of *C. clementina* (**b**) and *C. sinensis* (**c**). The colored blocks represent the different chromosomes in *C. clementina* (left) and *C. sinensis* (right). The genes evaluated for each species are shown in the corresponding chromosome

The establishment of synteny relations between species is an important tool to improve the understanding of genome evolution and the conserved biological functions among species [58]. To better understand the evolution of *C. clementina* and *C. sinensis* LRR-XII, we searched for syntenic blocks in the chromosomes. The similarity identified among LRR-XII gene sequences from one species in the genome of another species allowed us to identify conserved blocks in the *C. clementina* and *C. sinensis* chromosomes (Fig. 5). When analyzing the collinearity between both genomes, 25 syntenic blocks (SBs) were found between LRR-XII from *C. sinensis* and *C. clementina* (Fig. 5a). Of the 68 orthologous pairs previously identified by BBH and phylogeny, only 20 were also verified within these pairwise syntenic genes (Additional file 12). The different number of SBs identified resulted from a more stringent algorithm in this analysis. In addition, another analysis using the *Citrus* species independently found a total of 39 SBs in the chromosomes of *C. clementina* when evaluated with the 140 *C. sinensis* LRR-XII genes. A comparable number of 40 SBs was verified in the *C. sinensis* genome matching the 148 *C. clementina* LRR-XII genes. Some LRR-XII genes from both *Citrus* genomes matched more than one locus in the chromosomes and each locus was considered an independent SB. The number of LRR-XII genes that displayed similarity with the genome was of 26 of 148 LRR-XII genes from *C. clementina* and 25 of 140 LRR-XII genes for *C. sinensis* (Additional file 12). These numbers might be higher because we used a stringent analysis to increase the reliability of the results and avoid false positive SBs. In general, a reciprocal homology was observed in the SBs between *C. sinensis* and *C. clementina* chromosomes. We observed SBs distributed throughout almost all the chromosomes of the *Citrus* genomes (Fig. 5b-c). The highest number of SBs was found in chromosome 6 in both species, with 17 SBs for *C. clementina* and 16 for *C. sinensis*. The *C. sinensis* chromosome 2 matched the highest number of *C. clementina* LRR-XII, presenting homology with 6 sequences. For *C. clementina*, in addition to chromosome 2, chromosome 6 also exhibited the highest number of matches with *C. sinensis* LRR-XII, each of them displaying linkage with 6 genes in the corresponding chromosomes (Fig. 5b-c). Curiously, genes belonging to the same chromosome in one species matched SBs in different chromosomes from the other species, as in chromosome 2 from *C. clementina* and chromosomes 5, 8 and 10 from *C. sinensis* (Fig. 5b-c). In the case of chromosome 2 from *C. clementina*, one LRR-XII gene matched an SB in chromosome 9 from *C. sinensis,* while all the others matched SBs in chromosome 2. Chromosomes 10 or UN are particular because they are composed of sequences that were not assembled in the correct chromosomes. Therefore, it is an artifact from the genome assembly because the *Citrus* genome has only 9 chromosomes, thus the LRR-XII genes identified in this chromosome must be located in other genomic regions. On the other hand, genes in chromosomes 5 and 8 from the *C. sinensis* genome matched SBs in the same chromosome of *C. clementina* (Chr 8). This miscorrelation of some LRR-XII genes and SBs in the same chromosomes could be a result of chromosomal rearrangements in the genomes.

## Conclusions

This work provides the first comprehensive evolutionary analysis of the LRR-RLKs in *Citrus*. Lineage-specific expansion was observed in the *Citrus* LRR-XII group that might have occurred due to tandem duplications making the number of individuals larger compared to the majority of plant species. Considering the diverse number of pathogens affecting the domesticated citrus culture, the independent expansion of a defense-related group could be associated with an adaptive process related to plant-pathogen co-evolution. Our comparative data provided valuable information concerning these RLKs, opening new perspectives in the study of their function in diverse processes, such as development and defense responses in two worldwide important economic crops, specifically, sweet oranges and clementines.

## Methods

### Sequence database search

Predicted proteomes from *Citrus clementina* (Version 1.0, https://phytozome.jgi.doe.gov/pz/portal.html#!info?alias=Org_Cclementina), *Citrus sinensis* (Version CsiDB201301, http://citrus.hzau.edu.cn/orange/download/data.php) [26] and *Arabidopsis thaliana* (http://arabidopsis.org) were selected and downloaded. The InterProScan 4 package software (http://www.ebi.ac.uk/interpro/download.html) was used to identify different protein signatures in each proteome dataset [59]. To recover and analyze the dataset, we developed local relational databases for each included plant species. It allowed us to extract and interpret the large amount of data obtained in this work. In-house Perl scripts and Structured Query Language (SQL) queries were used to analyze data files during the database building and searching processes. Access to these local relational databases was implemented using DbVisualizer version 9.1.7 (http://dbvis.com/).

### Domain annotation and LRR-RLK retrieval

The LRR-RLK homologues were retrieved from the relational databases by searching for protein sequences with kinase, transmembrane and leucine-rich repeat domains. To recover the identifiers with KD, we considered Pkinase (PF00069) and Pkinase_Tyr (PF07714), according to the Pfam platform [60], as diagnostic domains. TMs were predicted from the TMHMM website (http://www.cbs.dtu.dk/services/TMHMM/) hosted at the Center for Biological Sequence Analysis, Technical University of Denmark. The prediction of transmembrane helices in the protein sequences were conducted according to the default parameters of version 2.0, without considering the old model option (version 1).

The following LRR diagnostic domains were searched: LRR_1 - Leucine Rich Repeat (PF00560), LRRNT - Leucine rich repeat N-terminal domain (PF01462), LRV - Leucine rich repeat variant (PF01816), LRRNT_2 - Leucine rich repeat N-terminal domain (PF08263), LRR_4 - Leucine rich repeats (2 copies) (PF12799), LRR_5 - Leucine rich repeats (6 copies) (PF13306), LRR_8 - Leucine rich repeat (PF13855), LRR_9 - Leucine-rich repeat (PF14580), LRRCT - Leucine rich repeat C-terminal domain (PF01463), LRR_2 - Leucine Rich repeat (PF07723), and LRR_3 - Leucine Rich repeat (PF07725). Only proteins containing LRRs, TM and KD were then considered to be putative LRR-RLK, and for this reason, At2g24130, which did not show TM, was not included in our analyses. Alternative splicing variants were excluded from our analysis.

### Kinase domain alignment and phylogenetic analysis

Sequences of conserved KDs from *Arabidopsis* and *Citrus* LRR-RLK proteins were extracted by an in-house Pearl script that consider KD coordinates annotation from the Pfam database. In addition, six human kinase protein sequences were used as an outgroup (Additional file 13). The KD sequences were aligned using MAFFT version 7 (http://mafft.cbrc.jp/alignment/software/) with G-INS-i strategy and default parameters [61]. The aligned sequences were visualized and manually refined using Jalview version 15.0 [62]. The proteins with a short length (<100 aa) or large inserted gap regions were removed. Gap-rich columns were further filtered using trimAl v.1.3 with the gappyout method [63]. To optimize the datasets for evolutionary analyses, the Decrease Redundancy tool, available as a resource at ExPaSy (www.expasy.org), was used to remove identical or distantly related sequences. The Decrease Redundancy parameters were set as 99 for “% max similarity” and 30 for “% min similarity”. Phylogenetic analyses were performed using the Maximum-likelihood method, as implemented in PhyML [64]. Twelve different evolutionary models (JTT, LG, DCMut, MtREV, MtMam, MtArt, Dayhoff, WAG, RtREV, CpREV, Blosum62 and VT) were tested using ProtTest 2.4 software [65]. The evolutionary model best fitting the data (best fit model) was determined by comparing the likelihood of the tested models according to the Akaike Information Criterion. A discrete gamma-distribution model with four rate categories plus invariant positions was assumed with the gamma parameter and the fraction of invariant positions was estimated from the data. Tree support values were estimated using approximate likelihood ratio test (ALRT), as implemented in PhyML. The ML trees were visualized and edited using the FigTree software (tree.bio.ed.ac.uk/software/figtree). The alignments are available at FigShare (10.6084/m9.figshare.3474752).

### Identification of the RD motif in the kinase domain

Identification of the *Citrus* RD motif in the catalytic loop from the LRR-XII kinase subdomain was performed using multiple expectation maximization for motif (MEME) suite web server using default parameters [66]. The kinases were classified as RD or non-RD according to the presence or absence of the Arg (R) in the conserved HRD motif, respectively.

### Chromosomal distribution of LRR XII

The genomic coordinates of each LRR-XII gene from *C. clementina* and *C. sinensis* were used to determine their distribution in the *Citrus* chromosomes. The coordinates were retrieved accessing the genome browser from each *Citrus* database. The MapChart graphical tool [67] was used to generate schematic diagrams to represent the LRR-XII gene positions in the chromosomes.

### LRR-XII orthologs and tandem duplicated paralogs

The identification of orthologous pairwise sequences among *Citrus* species was achieved through grouping in the phylogenetic tree and the BBH method. The Blastp searches were performed using all the *C. sinensis* and *C. clementina* LRR-RLK sequence proteins from group XII. For tandem duplicated paralogs searches, the results from Blasp were analyzed together with well-supported clades from the LRR-XII phylogenetic trees. The tandem duplicated paralogs were eligible when they formed the same clade and showed proximity in their chromosomal location. The identification of the LRR-XII gene clusters was performed from the arrangement of these genes in the chromosomes of each species. The LRR-XII genes were grouped in the same cluster if the genome location between two genes was within 200 kb in the chromosomes of *C. sinensis* and *C. clementina.*

### LRR-XII gene synteny identification

The synteny analyses were performed using *Sibelia* software. Although this tool was originally optimized to efficiently identify syntenic blocks between closely related microbial genomes [68], this tool was employed because the chromosome comparisons were restricted to a small gene family of *Citrus* species with evolutionary proximity.

The minimal nucleotide length considered in the syntenic block was adjusted to 1,000 pb. Iterative de Bruijn graphs were used to show the homology results found across the LRR-XII and the genomic regions in the chromosomes.

## Abbreviations

ACF, alternative catalytic function; ALRT, approximate likelihood ratio test; BBH, bidirectional best hits; BLAST, basic local alignment search tool; C, cysteine; CsiDB, citrus sinensis database; ECD, extracellular domain; EFR, Ef-Tu receptor; Ef-Tu, elongation factor thermo unstable; FLS2, flagellin sensing 2; FRK1, flg22-induced receptor-like kinase 1; G, glycine; HRD, histidine-arginine-aspartate; IOS1, impaired oomycete susceptibility 1; Kb, kilobase; KD, kinase domain; LRRCT, leucine-rich repeat C-terminal; LRRNT, leucine-rich repeat N-terminal; LRR-RLK, leucine-rich repeat receptor-like kinases; LRV, leucine-rich repeat variant; MAFFT, multiple alignment using fast fourier transform; MAMP, microbe-associated molecular pattern; MEME, multiple expectation maximization for motif elicitation; ML, maximum-likelihood; NBS, nucleotide binding site; PAMP, pathogen-associated molecular pattern; PRR, pattern-recognition receptor; R, arginine; RD, arginine-aspartate; RLCK, receptor-like cytoplasmic kinase; RLK, receptor-like kinase; S, serine; SB, syntenic block; SQL, structured query language; TAIR, the Arabidopsis information resource; TM, transmembrane; Un, unassigned; Y, tryptophan.

## Additional files

Additional file 1: Amino acid sequences from *Arabidopsis thaliana* LRR-RLKs. Dataset containing 209 LRR-RLK sequences obtained from http://arabidopsis.org/. (XLSX 137 kb) Additional file 2: Amino acid sequences from *C. clementina, C. sinensis* and *A. thaliana* kinase domains. The sequences were obtained by in-house Perl scripts using kinase coordinates from pfam database. (XLSX 108 kb) Additional file 3: Phylogenetic tree of LRR-RLKs from *Citrus clementina* and *Arabidopsis thaliana*. The phylogenetic tree was reconstructed with amino acid sequences from kinase domains by Maximum-likelihood method. The groups of LRR-RLK subfamily (I – XVI) are separated in different colors. Tree support values (aLRT) are indicated. (PDF 68 kb) Additional file 4: Phylogenetic tree of LRR-RLKs from *Citrus sinensis* and *Arabidopsis thaliana*. The phylogenetic tree was reconstructed with amino acid sequences from kinase domains by Maximum-likelihood method. The groups of LRR-RLK subfamily (I – XVI) are separated in different colors. Tree support values (aLRT) are indicated. (PDF 66 kb) Additional file 5: Phylogenetic tree of LRR-RLKs from *Citrus clementina*, *Citrus sinensis* and *Arabidopsis thaliana*. The phylogenetic tree was reconstructed with amino acid sequences from kinase domains by Maximum-likelihood method. The groups of LRR-RLK subfamily (I – XVI) are separated in different colors. (PDF 69 kb) Additional file 6: Complete list and classification of LRR-RLKs from *C. clementina, C. sinensis* and *A. thaliana. Citrus* sequences were categorized in 16 groups of LRR-RLKs. (XLSX 55 kb) Additional file 7: Pairwise list of orthologues LRR-XII subfamily members of *C. clementina* and *C. sinensis*. The colors represent the gene position in the same phylogenetic group (Fig. **2**). * represents genes in clusters. (XLSX 11 kb) Additional file 8: Classification of kinases from *C. clementina* and *C. sinensis* LRR-RLK as RD, non-RD and ACF. The kinases classification was done according to the presence or absence of the Arg (R) in the conserved HRD motif. (XLSX 13 kb) Additional file 9: Statistics of the tandem duplicated paralogues sequences from *C. clementina* LRR-XII. Comparative data generated by Blastp using all *C. clementina* LRR-XII. (XLSX 13 kb) Additional file 10: Statistics of the tandem duplicated paralogues from *C. sinensis* LRR-XII. Comparative data generated by Blastp using all *C. sinensis* LRR-XII. (XLSX 12 kb) Additional file 11: List of *C. clementina* and *C. sinensis* LRR-XII localized in cluster region. The chromosome distance of 200 kb was used as a reference to include the LRR-XII genes in the same cluster region. (XLSX 12 kb) Additional file 12: Syntenic blocks (SBs) of LRR-XII from *C. sinensis* and *C. clementina.* SBs were obtained from genome collinearity and independent homology analysis. (XLSX 18 kb) Additional file 13: Amino acid sequences of human kinase proteins. Sequences used as outgroup in the phylogenetic analysis with *Citrus* and *Arabidopsis* LRR-RLKs. (XLSX 10 kb)
