# Supplementary figures and images for: LRR-RLK family from two Citrus species: genome-wide identification and evolutionary aspects
Source: BMC Genomics. 2016 Aug 12;17:623. doi: 10.1186/s12864-016-2930-9 (PMC4982124; doi:10.1186/s12864-016-2930-9)

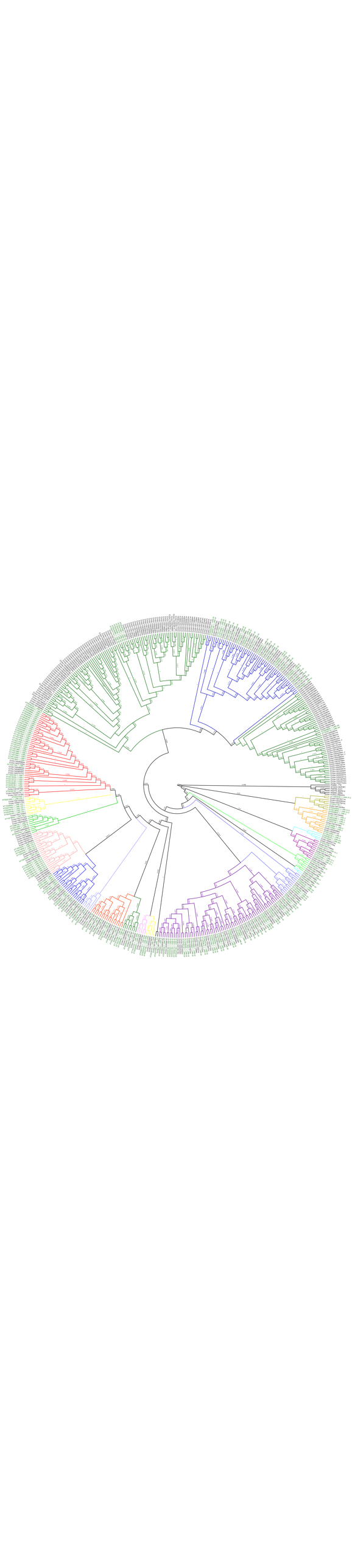

Supplement: Additional file 3: — Phylogenetic tree of LRR-RLKs from Citrus clementina and Arabidopsis thaliana. The phylogenetic tree was reconstructed with amino acid sequences from kinase domains by Maximum-likelihood method. The groups of LRR-RLK subfamily (I – XVI) are separated in different colors. Tree support values (aLRT) are indicated. (PDF 68 kb) [file 12864_2016_2930_MOESM3_ESM.pdf]

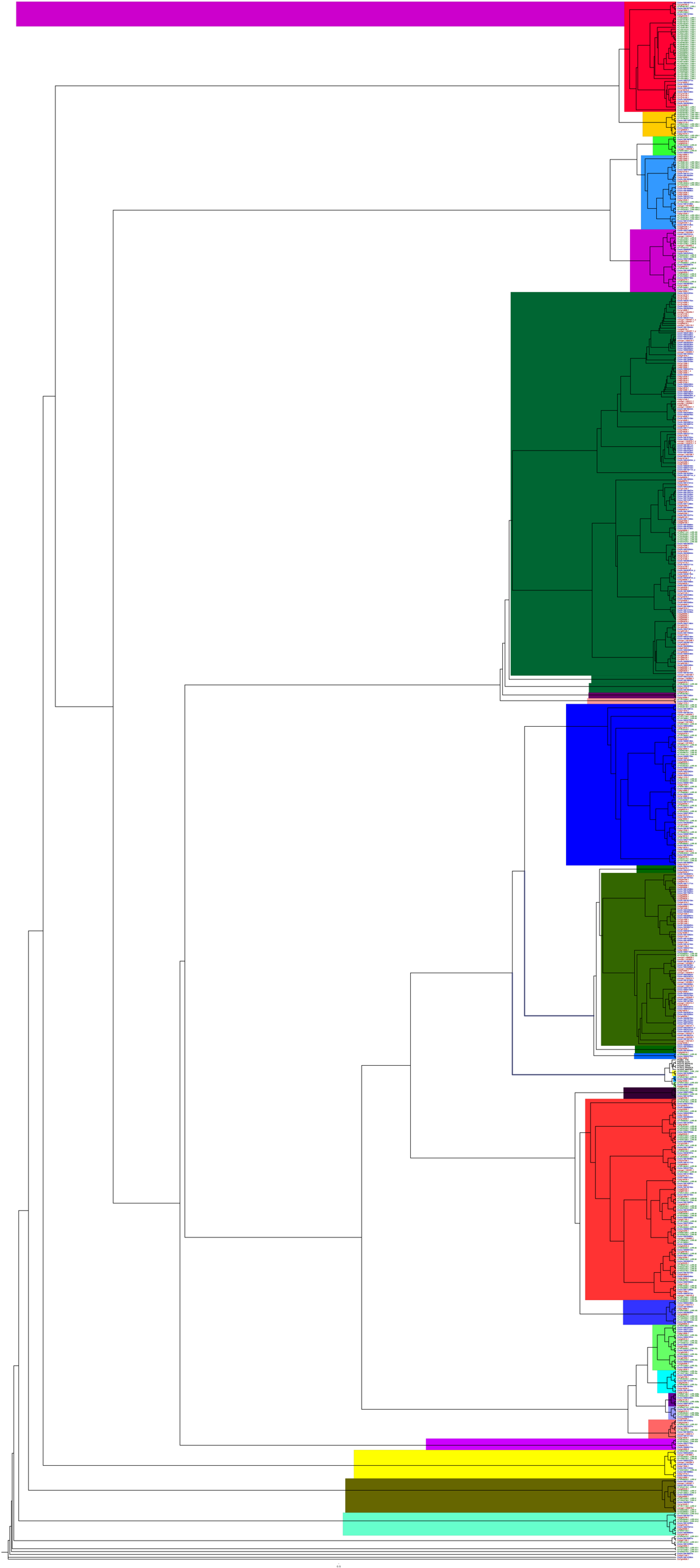

Supplement: Additional file 5: — Phylogenetic tree of LRR-RLKs from Citrus clementina, Citrus sinensis and Arabidopsis thaliana. The phylogenetic tree was reconstructed with amino acid sequences from kinase domains by Maximum-likelihood method. The groups of LRR-RLK subfamily (I – XVI) are separated in different colors. (PDF 69 kb) [file 12864_2016_2930_MOESM5_ESM.pdf]
